# Peptide mass fingerprinting of preserved collagen in archaeological fish bones for the identification of flatfish in European waters

**DOI:** 10.1098/rsos.220149

**Published:** 2022-07-27

**Authors:** Katrien Dierickx, Samantha Presslee, Richard Hagan, Tarek Oueslati, Jennifer Harland, Jessica Hendy, David Orton, Michelle Alexander, Virginia L. Harvey

**Affiliations:** ^1^ Department of Archaeology, University of York, Heslington YO10 5DD, York, UK; ^2^ Centre National de la Recherche Scientifique, University of Lille, Lille, France; ^3^ Archaeology Institute, University of the Highlands and Islands, Kirkwall, UK

**Keywords:** ZooMS, zooarchaeology, ichthyoarchaeology, fish remains, mass spectrometry, Pleuronectiformes

## Abstract

Bones of Pleuronectiformes (flatfish) are often not identified to species due to the lack of diagnostic features on bones that allow adequate distinction between taxa. This hinders in-depth understanding of archaeological fish assemblages and particularly flatfish fisheries throughout history. This is especially true for the North Sea region, where several commercially significant species have been exploited for centuries, yet their archaeological remains continue to be understudied. In this research, eight peptide biomarkers for 18 different species of Pleuronectiformes from European waters are described using MALDI-TOF MS and liquid chromatography tandem mass spectrometry data obtained from modern reference specimens. Bone samples (*n* = 202) from three archaeological sites in the UK and France dating to the medieval period (*ca* seventh–sixteenth century CE) were analysed using zooarchaeology by mass spectrometry (ZooMS). Of the 201 that produced good quality spectra, 196 were identified as flatfish species, revealing a switch in targeted species through time and indicating that ZooMS offers a more reliable and informative approach for species identification than osteological methods alone. We recommend this approach for future studies of archaeological flatfish remains as the precise species uncovered from a site can tell much about the origin of the fish, where people fished and whether they traded between regions.

## 1. Introduction

The North Sea is part of the Atlantic Ocean and is a shelf sea located for the most part on the European continental shelf with a surface area of around 575 000 square kilometers. This shallow and sandy/muddy sea is an ideal habitat for flatfish (Pleuronectiformes). Over 20 species of flatfish are reported from the North Sea, with around 12 species of modern day commercial interest [1].

Flatfish remains are difficult to identify to species using morphological analyses due to the lack of diagnostic criteria between taxa in many bones (e.g. [2–8]), which become even less useful when dealing with badly preserved archaeological bones. For example, since the 1990s, only 1–15% of all Pleuronectidae bones have been identified to species, while the remaining samples were categorized at family level (Pleuronectidae) or the *Pleuronectes platessa* Linnaeus 1758*/Platichthys flesus* (Linnaeus 1758)*/Limanda limanda* (Linnaeus 1758)-complex (plaice/flounder/dab, respectively) in some major zooarchaeological reports (e.g. [2,3,5–8]). This issue is more significant for vertebrae than cranial bones as there are even fewer diagnostic morphological features present in these elements that allow distinction between taxa (e.g. [4,9]). A similar problem is present within the Scophthalmidae family, whereby species rarely get identified (e.g. [5,7]). Within Soleidae *Solea solea* (Linnaeus 1758) (Dover sole) resembles *Pegusa lascaris* (Risso 1810) (sand sole), which are both present in the English Channel and the southern part of the North Sea [1].

Studying flatfish bones from archaeological sites around the North Sea area can help to better understand shifts in the environment, economy, fisheries, human diet and social status throughout history. Since these species complexes are difficult to identify, many questions remain unanswered about their exploitation and how it might have changed throughout time. Identifying species that are known from the more northern or southern areas from the North Sea, such as for example *Hippoglossus hippoglossus* (Linnaeus 1758) (halibut) and *S. solea*, respectively, can help to uncover historical environmental changes in the North Sea as well as potentially revealing trade in fish through time [10]. Differentiating species that can occur in freshwater environments, such as *P. flesus*, from marine species (such as *P. platessa* and *L. limanda*) can uncover changes in fisheries and the onset of intensive marine fish exploitation in Europe, the so-called ‘fish-event horizon’ which occurred during the medieval period (e.g. [11]). It is therefore important to identify archaeological remains of these fish to species wherever possible in order to understand the history of their exploitation. As flatfish fisheries continue to be of economic importance in modern times (e.g. [12,13]), insight into modern exploitation can help the management of the flatfish stocks. Species identification is therefore also of utmost importance when evaluating modern fisheries, and it has been shown that flatfish in the commercial food chain are often misidentified or mislabeled (e.g. [14–17]).

ZooMS (Zooarchaeology by Mass Spectrometry) uses peptide mass fingerprinting of collagen ‘Type I’ (hereafter ‘collagen’) preserved in bone tissue to help assign taxonomic identification [18–20]. ZooMS has been used to identify bones, teeth, skin and antlers of a wide variety of taxa (e.g. [19,21–33]), but also eggshells (e.g. [34,35]) and to identify human remains (e.g. [36–38]). There is a growing number of publications applying ZooMS to fish remains (e.g. [18,39–41]). The latest publications describing markers for Xiphiidae, Scombridae and Salmonidae, show the increasing utility of this technique to identify archaeological fish remains to genus and even species level [42–44]. Collagen of certain fish taxa consists of three collagen chains forming a triple helix: α1, α2 and α3. All these three chains differ from each other in their amino acid sequence, since all three are coded by different genes (COL1A1, COL1A2 and COL1A3). This makes certain fish collagen more diverse and more prone to show diagnostic markers between taxa, compared to that of all other vertebrates, which have only two different types of collagen chain (α1, α2) [39,45].

This study aims to improve flatfish identification through the use of a fast and affordable molecular alternative to traditional osteological methods by defining diagnostic peptide biomarkers in extracted flatfish collagen.

## 2. Material and methods

### 2.1. Collagen fingerprinting of Pleuronectiformes

#### 2.1.1. Sample selection

Modern Pleuronectiformes bones were sampled from museum and fresh specimens caught in the North Sea and surrounding areas and the Mediterranean Sea since the 1990s. The museum specimens (less than 31 years old) were taken from the collections held at the Royal Belgian Institute of Natural Sciences (RBINS) and the University of York Zooarchaeology Laboratory (YZL). Fresh fish from UK and Belgian shops were water macerated in an oven at 40°C for 2–3 days to retrieve their bones. Museum specimens preferably came from untreated bones, although warm-water maceration and cooking does not seem to have a large impact on the collagen quality [39]. Bones known to be treated with chemicals were avoided since the collagen could be damaged [39]. When sampling museum collections, vertebrae, branchial rays and fin rays were selected, as these are numerous in fish and contain little morphological information, reducing the impact of destructive analysis.

**Figure 1.**
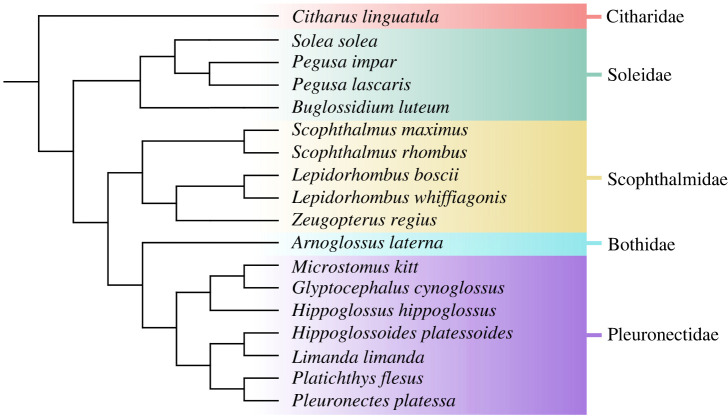
Cladogram showing the relations between the 18 species of Pleuronectiformes included in this study, based on Tinti *et al*. [46], Chanet [47] and Betancur *et al*. [48].

Eighteen flatfish species from five different families were sampled: Bothidae (*Arnoglossus laterna* (Walbaum 1792)), Citharidae (*Citharus linguatula* (Linneaus 1758)), Pleuronectidae (*Glyptocephalus cynoglossus* (Linneaus 1758), *Hippoglossoides platessoides* (Fabricius 1780), *Hippoglossus hippoglossus* (Linneaus 1758), *Limanda limanda* (Linneaus 1758), *Microstomus kitt* (Walbaum 1792)*, Platichthys flesus* (Linneaus 1758)*, Pleuronectes platessa* Linnaeus 1758), Scophthalmidae (*Lepidorhombus boscii* (Risso 1810)*, Lepidorhombus whiffiagonis* (Walbaum 1792)*, Scophthalmus maximus* (Linneaus 1758), *Scophthalmus rhombus* (Linneaus 1758)*, Zeugopterus regius* (Bonnaterre 1788)), and Soleidae (*Buglossidium luteum* (Risso 1810)*, Pegusa impar* (Bennett 1831)*, Pegusa lascaris* (Risso 1810)*, Solea solea* (Linneaus 1758)). Table 1 provides an overview and details of the specimens used for each species. Figure 1 shows a cladogram with the relations between the included species.

**Table 1.** List of modern specimens used for the ZooMS reference library. All samples were analysed using MALDI-TOF MS and a selection using LC-MS/MS.

genus	species	common name	museum collection	skeletal element	weight (mg)	LC-MS/MS
*Arnoglossus*	*laterna*	Med. scaldfish	RBINS A2-038-P-17	caudal vertebra	15.3	
*Arnoglossus*	*laterna*	Med. scaldfish	RBINS A2-038-P-18	caudal vertebra	20.4	x
*Arnoglossus*	*laterna*	Med. scaldfish	RBINS A4-020-P-02	caudal vertebra	21.3	
*Citharus*	*linguatula*	Spotted flounder	RBINS 24630	caudal vertebra	16.7	
*Citharus*	*linguatula*	Spotted flounder	RBINS 24631	caudal vertebra	20.3	x
*Citharus*	*linguatula*	Spotted flounder	RBINS 24632	caudal vertebra	18	
*Citharus*	*linguatula*	Spotted flounder	RBINS DCB842	caudal vertebra	28.5	
*Glyptocephalus*	*cynoglossus*	Witch	RBINS 91-017-P-55	caudal vertebra	27.7	
*Glyptocephalus*	*cynoglossus*	Witch	RBINS 91-017-P-56	caudal vertebra	21.8	
*Glyptocephalus*	*cynoglossus*	Witch	RBINS DCB359	fin ray	22.1	x
*Glyptocephalus*	*cynoglossus*	Witch	YZL 0902	caudal vertebra	15.3	
*Hippoglossoides*	*platessoides*	Long rough dab	RBINS 91-017-P-142	fin ray	25	x
*Hippoglossoides*	*platessoides*	Long rough dab	RBINS DCB767	caudal vertebra	26.4	
*Hippoglossoides*	*platessoides*	Long rough dab	RBINS DCB849	caudal vertebra	20.6	
*Hippoglossoides*	*platessoides*	Long rough dab	RBINS DCB850	caudal vertebra	31.6	
*Hippoglossus*	*hippoglossus*	Halibut	RBINS 91-017-P-2	caudal vertebra	31.5	x
*Hippoglossus*	*hippoglossus*	Halibut	RBINS 91-017-P-78	caudal vertebra	26.8	
*Hippoglossus*	*hippoglossus*	Halibut	RBINS A4-022-P-0005	fin ray	30.7	
*Hippoglossus*	*hippoglossus*	Halibut	RBINS DCB844	caudal vertebra	22.1	
*Hippoglossus*	*hippoglossus*	Halibut	YZL1970	branchiostegal ray	24.8	
*Hippoglossus*	*hippoglossus*	Halibut	YZL1970	part vertebra	35.1	
*Limanda*	*limanda*	Dab	RBINS 23876	fin ray	17.6	
*Limanda*	*limanda*	Dab	RBINS A2-028-P-0041	caudal vertebra	23.2	
*Limanda*	*limanda*	Dab	RBINS A4-002-P-0061	caudal vertebra	28.6	x
*Limanda*	*limanda*	Dab	YZL 0853	caudal vertebra	15.7	
*Microstomus*	*kitt*	Lemon sole	RBINS 23882	fin ray	22.4	
*Microstomus*	*kitt*	Lemon sole	RBINS A3-001-P-0062	caudal vertebra	34	
*Microstomus*	*kitt*	Lemon sole	RBINS A4-001-P-0088	caudal vertebra	24.6	
*Microstomus*	*kitt*	Lemon sole	RBINS A4-001-P-0091	fin ray	19.5	
*Microstomus*	*kitt*	Lemon sole	YZL 1963	caudal vertebra	31.2	x
*Platichthys*	*flesus*	Flounder	RBINS A2-028-P-61	caudal vertebra	29.5	
*Platichthys*	*flesus*	Flounder	RBINS A2-038-P-22	fin ray	16.7	
*Platichthys*	*flesus*	Flounder	RBINS A4-001-P-36	caudal vertebra	21.4	x
*Platichthys*	*flesus*	Flounder	YZL 1973	caudal vertebra	18.3	
*Platichthys*	*flesus*	Flounder	YZL 1974	caudal vertebra	18.8	
*Pleuronectes*	*platessa*	Plaice	RBINS 23806	fin ray	17.3	x
*Pleuronectes*	*platessa*	Plaice	RBINS 96-87-P-5	caudal vertebra	25.5	
*Pleuronectes*	*platessa*	Plaice	RBINS A2-057-P-27	caudal vertebra	19.6	
*Pleuronectes*	*platessa*	Plaice	YZL 1966	caudal vertebra	15.1	
*Pleuronectes*	*platessa*	Plaice	YZL 1967	fin ray	16.2	
*Pleuronectes*	*platessa*	Plaice	YZL 1968	caudal vertebra	24.9	
*Lepidorhombus*	*boscii*	Four-spot megrim	RBINS DCB773	caudal vertebra	9.7	x
*Lepidorhombus*	*whiffiagonis*	Megrim	RBINS 91-017-P-14	caudal vertebra	30.8	x
*Lepidorhombus*	*whiffiagonis*	Megrim	RBINS 91-017-P-26	caudal vertebra	20.4	
*Lepidorhombus*	*whiffiagonis*	Megrim	RBINS 91-017-P-59	fin ray	29.9	
*Lepidorhombus*	*whiffiagonis*	Megrim	RBINS A4-001-P-94	caudal vertebra	22	
*Scophthalmus*	*maximus*	Turbot	RBINS 91-017-P-98	caudal vertebra	30.5	
*Scophthalmus*	*maximus*	Turbot	RBINS A2-019-P-0047	caudal vertebra	33.3	
*Scophthalmus*	*maximus*	Turbot	RBINS A2-023-P-0002	fin ray	19.9	
*Scophthalmus*	*maximus*	Turbot	RBINS A2-052-P-0012	fin ray	26.1	x
*Scophthalmus*	*maximus*	Turbot	YZL 1962	caudal vertebra	24.3	
*Scophthalmus*	*maximus*	Turbot	YZL 1964	caudal vertebra	21.8	
*Scophthalmus*	*maximus*	Turbot	YZL 1965	caudal vertebra	19.4	
*Scophthalmus*	*maximus*	Turbot	YZL 1969	branchiostegal ray	27	
*Scophthalmus*	*maximus*	Turbot	YZL 1969	caudal vertebra	22.1	
*Scophthalmus*	*maximus*	Turbot	YZL 1969	fin ray	21.9	
*Scophthalmus*	*rhombus*	Brill	RBINS 23664	caudal vertebra	23	
*Scophthalmus*	*rhombus*	Brill	RBINS 23771	fin ray	25.8	x
*Scophthalmus*	*rhombus*	Brill	RBINS 24823	fin ray	31.9	
*Scophthalmus*	*rhombus*	Brill	RBINS A3-004-P-0016	caudal vertebra	19.6	
*Scophthalmus*	*rhombus*	Brill	YZL 1960	caudal vertebra	27.3	
*Scophthalmus*	*rhombus*	Brill	YZL 1961	caudal vertebra	20.4	
*Zeugopterus*	*regius*	Eckström's topknot	RBINS A2-019-P-0030	caudal vertebra	11	x
*Buglossidium*	*luteum*	Solenette	RBINS 23080	caudal vertebra	20.3	
*Buglossidium*	*luteum*	Solenette	RBINS 91-017-P-138	caudal vertebra	5.4	x
*Buglossidium*	*luteum*	Solenette	RBINS A4-020-P-03	caudal vertebra	6.7	
*Pegusa*	*impar*	Adriatic sole	RBINS DCB915	caudal vertebra	14.9	x
*Pegusa*	*lascaris*	Sand sole	RBINS A2-057-P-0049	caudal vertebra	20	
*Pegusa*	*lascaris*	Sand sole	RBINS A2-057-P-0051	caudal vertebra, fin ray	27.8	x
*Pegusa*	*lascaris*	Sand sole	RBINS A2-057P-0050	caudal vertebra	29.3	
*Pegusa*	*lascaris*	Sand sole	RBINS A3-004-P-0003	caudal vertebra	29.5	
*Solea*	*solea*	Dover sole	RBINS 91-017-P-90	caudal vertebra	21.1	
*Solea*	*solea*	Dover sole	RBINS 24857	fin ray	22.7	x
*Solea*	*solea*	Dover sole	RBINS A2-019-P-48	caudal vertebra	18.8	
*Solea*	*solea*	Dover sole	RBINS A2-036-P-28	fin ray	24.2	
*Solea*	*solea*	Dover sole	RBINS A4-001-P-133	caudal vertebra	27.3	
*Solea*	*solea*	Dover sole	YZL 1972	caudal vertebra	25.1	

#### 2.1.2. Collagen extraction

All laboratory analysis was undertaken at the University of York. Collagen was extracted from the fish bones using the acid insoluble protocol, adapted from Buckley *et al.* [19], which consists of the following steps: demineralization of the bone, gelatinization, digestion and purification. Demineralization of a small piece of bone, between 5 and 35 mg, occurred by adding 250 µl 0.6 M hydrochloric acid to the bone and leaving it at 4°C until the bone became demineralized and pliable, usually within 1 or 2 days. The acid was then removed and discarded. To remove any possible contaminants, such as humic acids, the remaining bone was rinsed once with 250 µl 0.1 M sodium hydroxide and three times with a 200 µl 50 mM ammonium bicarbonate (NH_4_HCO_3_) buffer of pH 8.0 (Ambic). The bone was then gelatinized in a heating block at 65°C in 100 µl Ambic for 1 h. A 50 µl aliquot of the supernatant was transferred to a new tube, to which 1 µl of 0.5 µg µl^−1^ trypsin was added, and the solution left overnight in a heating block at 37°C. Trypsin digests the collagen into strands of peptide at the C-terminal to arginine and lysine residues. After stopping the digestion by trypsin by adding 1 µl of 5% trifluoroacetic acid (TFA), the peptides were extracted and purified using 100 µl Pierce C18 ZipTips with washing (0.1% TFA and UHQ water) and conditioning (0.1% TFA in 50 : 50 acetonitrile and UHQ water) solutions, as per manufacturer's protocol.

#### 2.1.3. MALDI-TOF MS

Extracted collagen was spotted on a 384 steel target plate in triplicate. A 1 µl aliquot of every sample was spotted together with 1 µl of matrix solution (α-cyano-4-hydroxycinnamic acid). Each sample was externally calibrated against an adjacent spot containing a mixture of six peptides (des-Arg1-bradykinin *m/z* = 904.681, angiotensin I *m/z* = 1295.685, Glu1-fibrinopeptide B *m/z* = 1750.677, ACTH (1–17 clip) *m/z* = 2093.086, ACTH (18–39 clip) *m/z* = 2465.198 and ACTH (7–38 clip) *m/z* = 3657.929). The spots were air dried at room temperature. The samples were analysed using a Bruker Ultraflex III MALDI-TOF (matrix assisted laser desorption ionization-time of flight) mass spectrometer at the BioscienceTechnology Facility, University of York, with the following settings: ion source 25 kV; ion source 21.4 kV; lens voltage 9 kV; laser intensity 40–55%; and mass range 800–4000 Da. Peptide masses below 650 Da were suppressed.

#### 2.1.4. LC-MS/MS

LC-MS/MS was performed using a Thermo Scientific Orbitrap Fusion Tribrid housed at the Centre of Excellence in Mass Spectrometry, Chemistry Department, University of York on one specimen for each species (table 1). Data were acquired over 1 h acquisitions, with elution from a 50 cm PepMap and high resolution MS2 in DDA mode with the top 12 peaks selected for MS2 per scan.

Peptides were re-suspended in aqueous 0.1% TFA (v/v) then loaded onto an mClass nanoflow UPLC system (Waters) equipped with a nanoEaze *M/Z* Symmetry 100 Å C18, 5 µm trap column (180 µm × 20 mm, Waters) and a PepMap, 2 µm, 100 Å, C18 EasyNano nanocapillary column (75 µm × 500 mm, Thermo). The trap wash solvent was aqueous 0.05% (v:v) TFA and the trapping flow rate was 15 µl min^−1^. The trap was washed for 5 min before switching flow to the capillary column. Separation used gradient elution of two solvents: solvent A, aqueous 0.1% (v:v) formic acid; solvent B, acetonitrile containing 0.1% (v:v) formic acid. The flow rate for the capillary column was 300 nl min^−1^ and the column temperature was 40°C. The linear multi-step gradient profile was: 3–10% B over 7 min, 10–35% B over 30 min, 35–99% B over 5 min and then proceeded to wash with 99% solvent B for 4 min. The column was returned to initial conditions and re-equilibrated for 15 min before subsequent injections.

The nanoLC system was interfaced with an Orbitrap Fusion Tribrid mass spectrometer (Thermo) with an EasyNano ionization source (Thermo). Positive ESI-MS and MS2 spectra were acquired using Xcalibur software (v. 4.0, Thermo). Instrument source settings were: ion spray voltage, 1900 V; sweep gas, 0 Arb; ion transfer tube temperature; 275°C. MS1 spectra were acquired in the Orbitrap with: 120 000 resolution, scan range: *m/z* 375–1500; AGC target, 4e5; max fill time, 100 ms. The data-dependent acquisition was performed in topN mode using a selection of the 12 most intense precursors with charge states greater than 1. Easy-IC was used for internal calibration. Dynamic exclusion was performed for 50 s post precursor selection and a minimum threshold for fragmentation was set at 5e3. MS2 spectra were acquired in the Orbitrap with: 30 000 resolution, max fill time, 100 ms, HCD; activation energy: 32 NCE.

#### 2.1.5. Analysis

All spectra obtained from the MALDI-TOF MS were analysed using mMass software v. 5.5.0 [49]. The averaged spectrum was cropped between 800 and 4000 *m/z*. Data from the LC-MS/MS were searched against a local database with 151 published teleost fish collagen sequences obtained from NCBI Blast [50] using Mascot search engine (v. 2.8.0)[51] as follows: error tolerant; up to 1 missed cleavage; ±3 ppm peptide tolerance; ±0.01 Da MS/MS tolerance; 2+, 3+ and 4+ peptide charge; monoisotopic; Carbamidomethyl (C) as fixed modification; Oxidation (K) and Oxidation (P) as variable modifications. After the initial search, a decoy search was performed to verify the obtained amino acid sequences using the following settings: decoy; up to two missed cleavages; ±3 ppm peptide tolerance; ±0.01 Da MS/MS tolerance; 2+, 3+ and 4+ peptide charge; monoisotopic; carbamidomethyl (C) as fixed modification; oxidation (K), oxidation (M), oxidation (P) and deamidation (NQ) as variable modifications. The terminology used follows Unimod [52].

**Figure 2.**
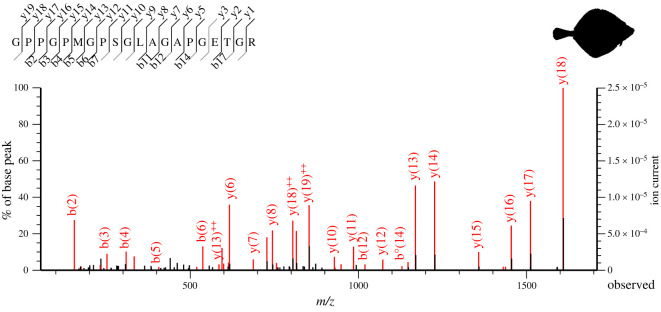
Example of a high-quality ion spectrum of the COL1ɑ1 817–836 peptide marker of *Pleuronectes platessa* with many *y*- and b-ions and high and isolated peaks as result of the Mascot search.

Mass peaks present in the MALDI-TOF MS data that differed between taxa were searched specifically in Mascot. If the score of the peptide given by Mascot was higher than the score for a false-positive match, the peptide was noted as a potential biomarker. Each high-scoring mass peak was checked for quality using the ion spectra given by Mascot. The criteria for a good quality fragment ion spectrum were: (i) many y- and b-ions and/or (ii) clear spectrum with high and isolated peaks (figure 2). Using the aligned collagen fish database with 151 sequences from NCBI Blast, the locus of the peptide from the LC-MS/MS could be found using BioEdit v. 7.2 [53]. The nomenclature used follows Brown *et al*. [54]. α1 and α3 collagen chains were differentiated following Harvey *et al.* [45]. The final selection of peptide biomarkers was made by choosing the minimum number of markers needed to distinguish between all species.

Flatfish collagen sequences were obtained *de novo* by scaffolding the peptide sequences obtained via Mascot. For each flatfish species, the whole collagen sequence of the best-matching database sample was cleaned up by removing all the peptides that did not have a score above the homology threshold provided by Mascot and copied into BioEdit. Using the predicted amino acid substitutions from Mascot, each peptide in the alignment was modified to match the most likely substitution. The non-matched part of the sequences were filled with the amino acid sequence of the taxonomically closest available species in NCBI Blast.

As all amino acid sequences of the biomarkers are obtained via LC-MS/MS and Mascot searches, no distinction could be made between isoleucine (Ile) and leucine (Leu) as these amino acids are isobaric (having the same mass). All possible Ile/Leu substitutions predicted by Mascot searches were therefore reported as leucine substitutions as standard. Substitutions between alanine (Ala) and serine (Ser) and between proline (Pro) and Ile/Leu result in a +16 Da mass shift, which is the same as when an amino acid oxidises. As Mascot cannot distinguish between these cases, the most likely amino acid sequence was selected out of the options Mascot provided, based on the probability scores of the different amino acids, the quality of the ion spectra, and the principle of parsimony using the sequence of the most closely related species.

### 2.2. Archaeological application

**Figure 3.**
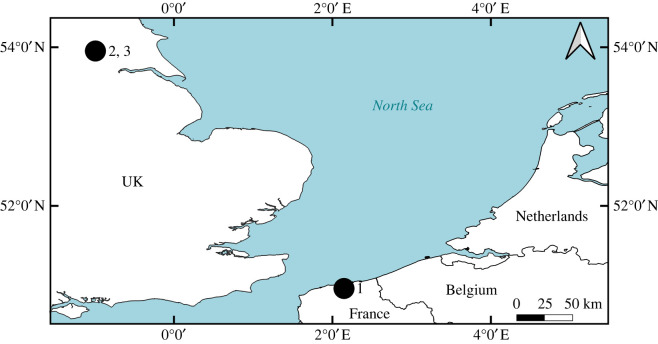
Map of the southern North Sea basin with the location of the three archaeological sites. 1: Barreau Saint-George-Desserte ferroviaire; 2: 16–22 Coppergate; 3: Blue Bridge Lane.

A total of 202 archaeological flatfish bones were selected from three archaeological sites from the North Sea basin: Barreau Saint-George-Desserte ferroviaire in northern France (*n* = 92); 16–22 Coppergate (*n* = 96) and Blue Bridge Lane (*n* = 14), both from York in the UK (figure 3). The samples were morphologically identified to family level according to diagnostic morphological criteria for each element as published in Wouters *et al.* [4] for Pleuronectidae and following comparisons with reference specimens of Pleuronectidae and Scophthalmidae using the fishbone collection at the University of York. From each context, one sample from each potentially different individual was selected, which was determined by the species identification, element representation and the estimated size of the individual fish. A substantial quantity of fish bones were uncovered at each of these sites which have been well reported in the literature: Oueslati [8] for Barreau Saint-George and Harland *et al.* [7] for both York sites. Table 2 summarizes the reported flatfish remains from each of the three sites per taxon and period. Original morphological identifications were available for 75 of the Coppergate bones and all (*n* = 14) of those from Blue Bridge Lane.

**Table 2.** Reported flatfish remains per taxon as identified morphologically and per period (CE) from Barreau Saint-George-Desserte ferroviaire (BSG) by Oueslati [8], and 16–22 Coppergate and Blue Bridge Lane by Harland *et al.* [7]. ‘a’ indicates that the species might be present, but identification was not confirmed.

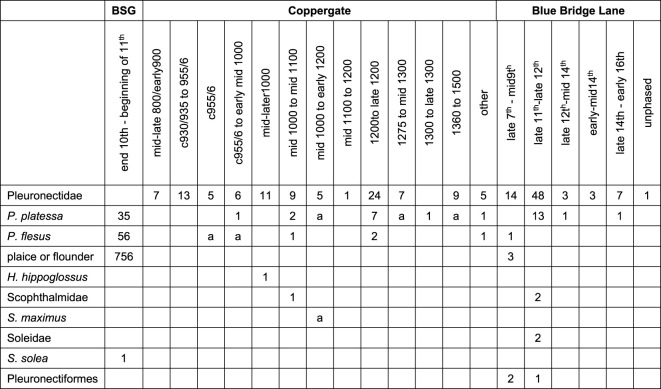

Barreau Saint-George-Desserte ferroviaire (50°58′27.8″ N, 2°10′7.6″ E) is located in the city of Saint-George sur-L'Aa in northern France, close to the coast and connected to the sea by the river Aa. The site dates from the end of the tenth century to the beginning of the eleventh century CE. The abundant fish remains from this site were identified as mostly of Pleuronectidae, a single *S. solea* and some Gadidae [8]. 16–22 Coppergate (53° 57′ 27.4″ N, 1° 4′ 51.5″ W) is situated in the city centre of inland York, UK, between the rivers Ouse and Foss. A large diversity of fish species have been reported [7] with *Anguilla anguilla* (Linnaeus 1758), Clupeidae, Cyprinidae, *Esox lucius* Linnaeus 1758 and Salmonidae being the more common species in the Anglo-Scandinavian periods (seventh–eleventh century CE), while Gadidae and Pleuronectidae become more abundant during the High and Late medieval periods (eleventh–fifteenth century CE) [7]. The selected samples from this site date from the Roman period (first–fourth century CE) to the Late Medieval period (thirteenth–fourteenth century CE). Blue Bridge Lane (53°57′5.6″ N, 1°4′34.5″ W) lies south of the walled city centre of York at Blue Bridge Lane on the east bank of the river Ouse, at its confluence with the river Foss. *Clupea harengus* Linnaeus 1758 is the most abundant species in this site, but also *A. anguilla*, *E. lucius*, Cyprinidae and Gadidae are common in certain phases [7]. The selected samples from Blue Bridge Lane date from the seventh century to the sixteenth century CE.

More than half (*n* = 113) of the archaeological samples were analysed following the same protocol as described above for the modern reference samples (see electronic supplementary material, table S9 for details). The remaining samples (*n* = 89) were analysed following a different protocol so that the extracted protein from these selected samples was also available for stable isotope analysis, which requires a greater amount of collagen. Here, 50–500 mg bone was demineralized with 0.4 M HCl at 4°C until the hydroxyapatite was dissolved. The remaining bone was rinsed with ultra-pure water and gelatinized by adding 8 ml of 0.001 M HCl to each sample and placing them in a heating block at 70°C for 24–48 h. An Ezee-filter was used to remove insoluble debris from the samples before freeze drying for 48 h. ZooMS was performed by dissolving approximately 1 mg of extracted collagen in Ambic solution, adding 1 µl trypsin and leaving the samples overnight at 37°C. The samples were then filtered using ZipTips, plated and analysed on the MALDI-TOF MS following the procedure described above. Each sample was identified by searching for the diagnostic masses from the selected peptide biomarkers on the mass spectra and by matching them to the mass spectra from the reference samples.

### 2.3. Data deposition

Datafiles of the MALDI-TOF MS spectra, LC-MS/MS raw and mgf files, and MZID files of the Mascot query against the collagen database of the reference samples and the MALDI-TOF MS spectra of the archaeological samples were deposited on Dryad and can be accessed by following this link: https://doi.org/10.5061/dryad.5qfttdz7f.

## 3. Results

### 3.1. Taxon resolution

Each of the 18 species included in this study were found to have a unique combination of peptide biomarkers, confirming that European flatfish can be identified to species using collagen peptide fingerprinting. All species can be identified using only eight different peptide biomarkers: COL1ɑ1 817–836, COL1ɑ1 934–963, COL1ɑ2 625–648, COL1ɑ2 658–687, COL1ɑ2 688–704 and COL1ɑ2 757–789 for all species, and additionally COL1ɑ3 889–909 for Scophthalmidae and COL1ɑ2 991–1027 for *Pegusa* sp. The peptide markers and their corresponding masses are summarized in table 3 and the differences between the homologous sequences are detailed in electronic supplementary material, tables S1–S8. Each time, *Pleuronectes platessa* is used as the base sequence whenever possible as this is the taxonomic type species of the order. In one case, *Platichthys flesus* is used as the base sequence, as this is the closest related species to *P. platessa*. No sequences were recovered for peptide ɑ1 934 in *Z. regius* and *C. linguatula*, for ɑ2 658 in *G. cynoglossus* and *A. laterna*, for ɑ2 688 in *P. platessa* and for ɑ2 757 in *A. laterna,* possibly because their sequences did not match any of the sequences in the custom database. Several peptide biomarkers did not show on the MALDI-TOF spectra, but did provide a result when searching using the LC-MS/MS data, probably because not all peptides are charged and detected by the MALDI-TOF MS; these are put between brackets in table 3. In several peptide biomarkers, oxidations of proline or other post-translational modifications were noted for some species, resulting in a mass shift compared with the expected mass based on the amino acid substitutions for that species. Oxidations were also noted if they were seen in the MALDI-TOF MS spectra and uncovered using the Mascot search. The collagen mass fingerprint spectra of each species (electronic supplementary material, figures S1–S18) and the ion spectra of each peptide biomarker for each species (electronic supplementary material, figures S19–S127) can be found in the electronic supplementary material.

**Table 3.** List of the selected collagen peptide biomarkers with corresponding mass peaks (*m/z*) per Pleuronectiformes species. Mass peaks that are not or not always visible in the mass spectra are noted between brackets.

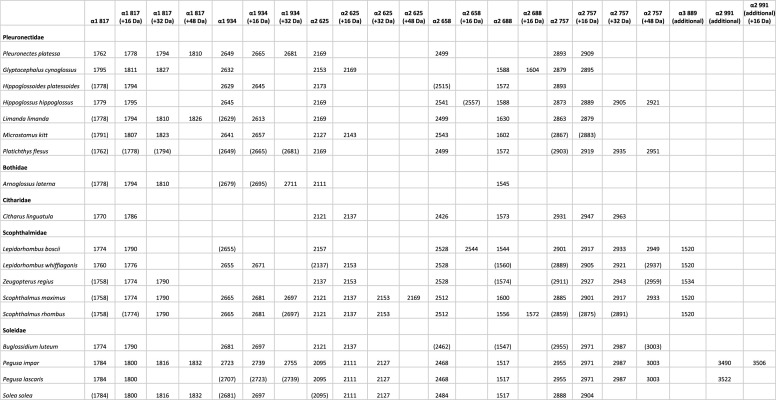

#### 3.1.1. Pleuronectiformes

All flatfish share a peptide peak at *m/z* 1878 (GFPGTPGLPGIKGHR) of COL1ɑ1 76–90, but this mass peak also seems to be shared with other common species from the eastern Atlantic area such as *E. lucius*, *Melanogrammus aeglefinus* (Linnaeus 1758), Cyprinidae and *Gadus morhua* Linnaeus 1758*.* No single distinct peptide marker was found that is unique to flatfish, but rather it is the combination of multiple biomarkers that distinguishes a particular species. All flatfish species analysed here can also be easily distinguished from other published fish species using the peptide biomarkers described in Harvey *et al.* [41], Rick *et al.* [42], Korzow Richter *et al.* [43] and Buckley *et al*. [44], as these show different combinations of mass peaks, which match with none of the flatfish.

#### 3.1.2. Pleuronectidae

**Figure 4.**
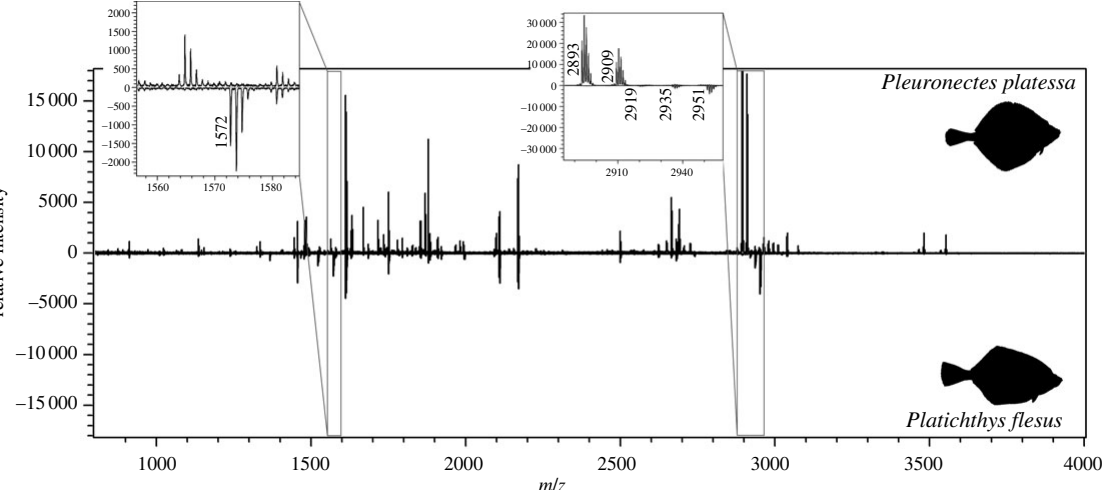
Collagen fingerprint comparison between *Pleuronectes platessa* (top) and *Platichthys flesus* (bottom) with details of the peptide markers α2 688–704 (left) and α2 757–789 (right).

No distinct peptide was found that is unique to the Pleuronectidae. Several Pleuronectidae species share the same sequence and mass for some of the selected peptide biomarkers. Interestingly, *Microstomus kitt,* whose placement as a Pleuronectid genus is confirmed by mtDNA and nDNA studies (e.g. [48,55]), has no mass or sequence shared with any of the other Pleuronectidae, indicating that this species is more differentiated and therefore likely to be more evolutionary diverged from the other Pleuronectidae. This case confirms the potential of using the amino acid sequence of collagen as a tool for the phylogenetic mapping of species, as described in Harvey *et al*. [45]. The other Pleuronectidae can be distinguished from each other by combining several of the selected biomarkers. Crucially, the osteologically similar species *P. platessa* and *P. flesus* can be distinguished by just two peptide biomarkers, illustrated in figure 4.

#### 3.1.3. Scophthalmidae

**Figure 5.**
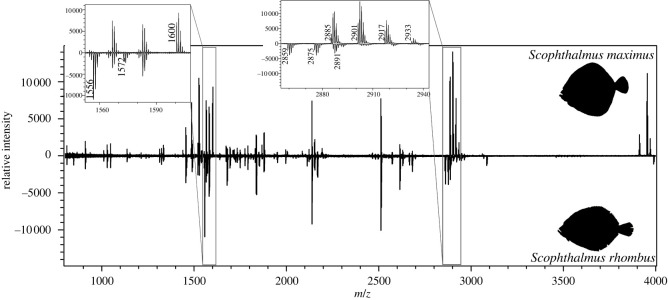
Collagen fingerprint comparison between *Scophthalmus maximus* (top) and *S. rhombus* (bottom) with details of the peptide markers α2 688–704 (left) and α2 757–789 (right).

All Scophthalmidae share the same sequence for ɑ2 658, although *Scophthalmus* sp. have a lower mass than *Zeugopterus* and *Lepidorhombus* sp. due to the lack of an oxidative modification. Each Scophthalmidae species has a unique sequence for ɑ2 757. Additionally, ɑ1 817, ɑ1 934, ɑ2 625, ɑ2 688 and ɑ3 889 provide diagnostic information for this family. Several masses described in the *Scophthalmus* sp. here, were already noted by Harvey *et al.* [41] for these species: *m/z* 1600, *m/z* 1774/1790, *m/z* 2137 and *m/z* 2665/2681. For *S. rhombus*, however, no peak at *m/z* 1600 was observed in this study and the peak at *m/z* 1223 described by Harvey *et al.* [41] for *S. maximus* was not observed in the specimens used for this study, while most *Scophthalmus* sp. showed a peak at *m/z* 1239. One *S. rhombus* did show a peak at *m/z* 1223. The osteologically similar *S. maximus* and *S. rhombus* can be distinguished by two peptide biomarkers, illustrated in figure 5.

#### 3.1.4. Soleidae

**Figure 6.**
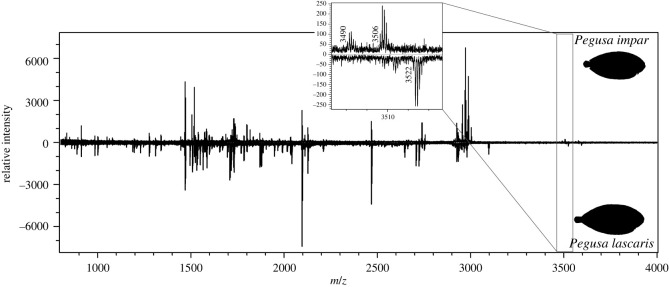
Collagen fingerprint comparison between *Pegusa impar* (top) and *P. lascaris* (bottom) with details of the peptide markers α2 991–1027.

*Pegusa* sp. and *S. solea* share the same sequence for five of the seven selected biomarkers. *Buglossidium luteum* often has a unique amino acid sequence for the markers. *Pegusa* sp. and *S. solea* can be distinguished using ɑ1 934 and ɑ2 757. *Pegusa impar* shows a peak at 1517 *m/z* from ɑ2 688 in the mass spectrum, but in the reference sample from this study it also showed a slight peak at 1516 *m/z* from COL1ɑ1 076–090 and COL1ɑ1 889–906. *Pegusa impar* and *P. lascaris* do not have different peptide biomarker sequences but do however show differences in their mass spectra, albeit for two markers (ɑ1 934 and ɑ2 991) only with a ±16 Da difference, possibly caused by oxidation, of which only the latter marker distinguishes the species (figure 6).

#### 3.1.5. Other taxa

*Arnoglossus laterna* and *Citharus linguatula*, both being the only representatives of their families in this study, have distinct masses and sequences for several of the markers, which are not shared by any of the other species.

#### 3.1.6. Possible issues in data analysis

In some cases, there are overlapping mass peaks visible in the peptide mass fingerprints, which can cause potential confusion when using the selected peptide biomarkers to identify species. For some of the diagnostic masses, another species can show a peak at the same mass (isobaric). In these cases, this peak originates from a different collagen peptide than the diagnostic one (table 4).

**Table 4.** List of isobaric masses and peptide markers found with their sequences and the peptide biomarkers with the same masses.

species	mass	sequence	locus	confusion with locus	confusion with species	remarks
*C. linguatula*	1534	R.GNPGAAGAAGAQGPIGPR.G	a2 502	a3 889	*Z. regius*	
*S. rhombus*	2111	K.GSPGAEGPSGASGLPGPQGIAGSR.G	a1 757	a2 625	*S. solea, A. laterna, Pegusa impar, Pegusa lascaris*	
*M. kitt*	2665	?	?	a1 934	*S. maximus, S. rhombus, P. flesus, P. platessa*	only in 2 samples, no match in Mascot
*G. cynoglossus*	2863	R.GLTGPIGLPGSAGSTGDKGEPGAAGPVGPGGAR.G	a1 586	a2 757	*L. limanda*	
*P. platessa*	2947	R.GVMGPTGPVGAPGKDGDVGAQGQSGPAGPAGER.G	a1 421	a2 757	*P. lascaris*	
*S. rhombus*	2947	R.GPPGPAGSSGPQGFTGPPGEPGEAGASGPMGPR.G	a1 010	a2 757	*P. lascaris*	
*S. maximus*	2947	R.GPPGSPGSSGPQGFTGPPGEPGEPGASGPMGSR.G	a3 010	a2 757	*P. lascaris*	
*S. solea*	2947	R.GPPGPAGSSGPQGFTGPPGEPGEAGAAGPMGPR.G	a1 010	a2 757	*P. lascaris*	
*P. platessa*	2947	R.GPPGPSGSSGPQGFTGPSGEPGEPGAAGPMGPR.G	a1 010	a2 757	*P. lascaris*	

### 3.2. Archaeological sample identification

Out of the 202 analysed archaeological flatfish bones, 99.5% (201 of 202) of the samples provided a clear mass spectrum suitable for species identification. Out of these 201 successful spectra, 196 were identified as a flatfish species. Only one sample failed to provide a mass spectrum of adequate quality to allow taxonomic identification, most likely due to a lack of preserved collagen. Most of the samples analysed were identified to *P. platessa* and *P. flesus*, with a few examples each of *L. limanda* and *S. maximus* (table 5; electronic supplementary material, figures S128–131). Detailed information on the context, dating, estimated size of the fish, skeletal element, original identification, protocol and ID markers used for each sample can be found in electronic supplementary material, table S9. Due to the lack of labelling, it was not possible to match any ZooMS samples from Barreau Saint-George and 21 from Coppergate to osteologically identified samples from previous reports.

**Table 5.** Overview of the number of samples identified to species by ZooMS from the three archaeological sites.

species	Barreau-Saint George (FR)	Coppergate, York (UK)	Blue Bridge Lane, York (UK)	total
*Pleuronectes platessa*	34	57	10	101
*Platichthys flesus*	58	24	3	85
*Limanda limanda*	0	5	1	6
*Scophthalmus maximus*	0	4	0	4
**total identified species**	**92**	**90**	**14**	**196**
Failed	0	1	0	1
Unknown species	0	5	0	5
**Total per site**	**92**	**96**	**14**	**202**

Table 6 compares the success ratio of ZooMS with the osteological identifications performed previously on these sites by other authors. Analysis through ZooMS resulted in species identifications for between 93.8% and 100% of the flatfish bones from each site, where only 10.9% to 15.7% of flatfish bones could be identified to species using traditional methods [7,8]. The ratio between *P. platessa* and *P. flesus* was similar for both ZooMS and the zooarchaeological report on Barreau Saint-George [8], while the amount of *P. flesus* found using ZooMS was higher than was reported from both York sites [7] (electronic supplementary material, table S10). Somewhat unexpectedly, the *L. limanda* and *S. maximus* that were identified through ZooMS were not reported in the previous morphological assessments.

**Table 6.** Comparison of the identification success rate of ZooMS applied to the selected samples compared with the success rate of osteological identifications as published in the zooarchaeological reports for the three sites. Data from the zooarchaeological reports taken from Harland *et al.* [7] and Oueslati [8]. Higher taxon level means any osteological identification to genus, family or order.

	identified using osteology		identified using ZooMS	
	number	percentage	number	percentage
*Barreau Saint-George*				
NISP	848		92	
higher taxon	756	89.16%		
species level	92	10.85%	92	100%
*Coppergate*				
NISP	120		96	
higher taxon	103	85.83%		
species	17	14.17%	90	93.75%
*Blue Bridge Lane*				
NISP	102		14	
higher taxon	86	84.31%		
species	16	15.69%	14	100%

A total of 74 Coppergate and 14 Blue Bridge Lane specimens were available for direct comparison of the original attributions with those derived from ZooMS (electronic supplementary material, table S11). Of the 19 samples identified to species osteologically, only three were misidentified according to the ZooMS identifications. Approximately a fifth of specimens were successfully identified to species osteologically, and most of these were cranial elements, which naturally have more variation between species and are thus easier to identify by morphology. Most of the morphological family level identifications were successful: 69%; with ZooMS then providing further refinement to species level. These were mostly vertebrae, as they are morphologically very difficult to distinguish to species. Six Coppergate bones were morphologically misidentified in some way: three cranial elements were incorrectly identified as *P. platessa* when they were *P. flesus* or vice versa; one was incorrectly identified as Pleuronectidae when it was Scophthalmidae; and two were identified as Pleuronectidae but ZooMS identified them as an unknown fish from the Perciformes order. One vomer was morphologically identified as Scophthalmidae, with a note that the specimen was unusually large and difficult to identify; ZooMS identified this as *P. platessa*. One originally identified bone failed to provide a usable spectrum for ZooMS identification.

Within the York sites, there is a clear switch in dominant flatfish species throughout the medieval period (table 7). During the early Medieval period/Anglo-Scandinavian period (seventh–mid/late eleventh century CE), *Platichthys flesus* is the dominant species within the samples analysed for both case studies in York, while during the High and Late medieval periods (mid-eleventh–late twelfth/early thirteenth and twelfth–sixteenth century CE) *Pleuronectes platessa* becomes the most abundant flatfish species.

**Table 7.** Distribution of *Pleuronectes platessa* and *Platichthys flesus* samples per larger time period of Coppergate and Blue Bridge Lane.

period (century CE)	*Pleuronectes platessa*	*Platichthys flesus*
7^th^ - mid 10^th^	2	15
Mid 10^th^ - mid/late 11^th^	2	4
Mid 11^th^ - late 12^th^/early 13^th^	18	3
12^th^ - 16^th^	45	3

One bone, initially selected for analysis as it resembled *S. solea*, turned out to be a *C. harengus* after matching it with the spectra published by Harvey *et al.* [41]. Three samples were similar to each other in their mass spectrum and morphologically resembled *Perca fluviatilis*, matching tentatively with the published spectrum from this species by Harvey *et al.* [41]. The fifth sample did not match any known spectrum, but does show some mass peaks also present in Pleuronectiformes.

## 4. Discussion

### 4.1. Species identification of flatfish using ZooMS

Collagen fingerprinting by mass spectrometry allows straightforward distinction between multiple species of flatfish (Pleuronectiformes) from European waters, especially those of the North Sea. Flatfish species that are frequently reported at archaeological sites and that are able to reach sizes larger than 20 cm SL (standard length), making them interesting for commercial purposes, were included in this study. As not all of the smaller Pleuronectiformes species in European waters were included, mostly due to a lack of access to samples during the coronavirus pandemic, caution is advised when applying this technique to bones from smaller sized fish. Additional species from the North Sea and surrounding areas, such as *Microchirus variegatus* (Donovan 1808), *Zeugopterus norvegicus* (Günther 1862) and *Z. punctatus* (Bonnaterre 1788) from the North Sea and *Reinhardtius hippoglossoides* (Walbaum 1792) from the North Atlantic, should be included in future studies to make more definitive conclusions, especially when trade from more southern or northern Atlantic areas or even the West-Atlantic and Mediterranean is suspected. Based on the results presented here, it can be expected that different genera of flatfish can easily be distinguished using several peptide markers. Within the same genus, however, there might be more difficulties to differentiate between species, depending on the time passed since the divergence of the species, which is correlated to the number of amino acid substitutions of collagen [40].

Notably, six of the eight selected biomarkers for flatfish were used in previous studies as good markers to distinguish between other fish taxa: ɑ1 688, ɑ1 817, ɑ1 934, ɑ2 625, ɑ2 658, ɑ2 688 and ɑ2 757 [41–44]. This could indicate that these specific locations in the collagen sequence are more prone to amino acid substitutions than other regions of the protein, resulting in clear differences between taxa as they evolutionary diverge from each other. The proposed biomarker for *Scophthalmus* sp. at *m/z* 1223/1239 found by Harvey *et al.* [41], however, was not found consistently in this dataset. Both masses can occur in both species as well as in other flatfish, but are just as often absent from *Scophthalmus* sp. Searching for these masses using Mascot did not return any sequences for *S. maximus* and *S. rhombus*. These peptide peaks were therefore not selected as diagnostic biomarkers for flatfish species.

The one available sample of *Z. regius* provided low quality MALDI-TOF and LC-MS/MS data. Since there is only one sample for this species, as for *P. impar* and *L. boscii*, the presence of mass peaks in fingerprints could not be verified and must be used cautiously until more samples are analysed that show the observed biomarkers to be species-specific and to occur consistently in all conspecifics.

*Pegusa impar* and *P. lascaris* only differ in their mass spectra by a mass shift caused by oxidation, which is not a reliable discriminator, meaning that archaeological samples cannot be identified to the correct species with certainty using ZooMS. As *P. impar* occurs only in the Mediterranean and the southern eastern Atlantic [56], this species could be excluded in some cases when dealing with fish remains from the Atlantic region. However, we cannot exclude the potential of fish being traded between regions. In the Mediterranean region, however, both *Pegusa* sp. can occur as well as many other Soleidae [56].

As some species show isobaric peptides with some of the selected peptide biomarkers of other species, there could potentially be some confusion when trying to identify species using MALDI-TOF MS spectra. For each species for which confusion with another species can happen due to isobaric peptides, only one diagnostic mass seems to be involved, meaning that the other diagnostic masses should not be affected by this. It is therefore advised to use as many of the selected peptide biomarkers as possible when identifying and not to rely on solely one biomarker for each species. Furthermore, it is important to know that some of the proposed biomarkers can be of low intensity in the mass spectra, but that their presence/absence is more important than their intensity for identification purposes. The use of a reference mass spectrum, such as those provided in the electronic supplementary material, to compare against a sample's mass spectrum is also advised.

With certain Actinopterygii species having a diversified α3 collagen chain, the gene for which originates from the gene coding for the α1 chain, the sequences and therefore the mass from the corresponding locus in both chains could be either the same or different [39,41]. This was noted for COL1ɑ1 76–90, which has the same sequence and mass in Pleuronectiformes as COL1ɑ3 76–90. *Esox lucius* and *Gadus morhua,* two European species for which sequence data from the collagen database on Blast was available for the isobaric mass peak, did not have the same sequence for COL1ɑ3 76–90 due to amino acid substitutions. The ɑ3 can therefore provide more variability in certain taxa as it can be diversified, but could potentially also cause some issues interpreting the mass peaks of peptides when they are isobaric.

### 4.2. Archaeological identification and interpretation

As shown by the three archaeological case studies presented here, ZooMS provides objective, reliable and high resolution identification of the species assemblage of flatfish remains compared with traditional osteological methods. As such it has the potential to uncover the hidden diversity of flatfish in archaeological assemblages that would otherwise go undetected.

The low diversity and relative frequencies of flatfish species found in these three case studies from two different geographical regions confirms the general conclusions from zooarchaeological studies of flatfish around the North Sea area. These indicate that the majority of flatfish remains uncovered represent only a few species, dominated by *P. platessa* and *P. flesus* with occasional finds of *L. limanda*, *H. hippoglossus*, *M. kitt*, *S. solea*, *S. maximus* and *S. rhombus*. A surprising number of *L. limanda* and *S. maximus* were, however, uncovered using ZooMS. At both sites in York, the presence of *L. limanda* was not mentioned in the zooarchaeological report by Harland *et al.* [7]. This suggests that some of the less frequently reported species might be more common in the zooarchaeological assemblages than previously understood. With collagen mass fingerprinting, these species might become more visible than relying solely on osteological methods.

*Platichthys flesus* and *Pleuronectes platessa* are common flatfish species found in the northeast Atlantic. Both species use shallow coastal or estuarine environments for spawning, but when the fish get larger, *P. flesus* is more likely to remain in the estuary or coastal regions, while *P. platessa* moves out to more open marine environments [57]. Adult *Platichthys flesus* is also found in estuaries, rivers and seas that have a lower salinity than the North Sea and Atlantic Ocean, while adult *P. platessa* seems to be absent or much less common in these habitats (e.g. [58–60]. *Platichthys flesus* also appears to have a preference for specific locations in an estuarine and riverine environments based on its size, with the smaller *P. flesus* more common upstream, while larger *P. flesus* are more common downstream (e.g. [61,62])

The large proportion of *P. flesus* in Barreau Saint-George is therefore noticeable. Given the small estimated size of these fish (see electronic supplementary material, information), this would suggest that the juvenile *P. flesus* were exploited in estuaries. As it is thought that flatfish were mostly targeted for local consumption in this site [8], a nearby exploitation of small flounder would be practical. Samples from *P. platessa* on the other hand, seem to have come from both small and larger individuals, which are more likely to have been captured in more coastal waters.

At both York sites, a dominance of *P. flesus* within the ZooMS samples is apparent in the Anglo-Scandinavian periods (*ca* seventh–eleventh century CE), while *P. platessa* became the most abundant species in the High and Late Medieval Periods (*ca* eleventh–sixteenth CE). A slight dominance of *P. platessa* during the twelfth–fourteenth century CE in Coppergate and Blue Bridge Lane was noted by Harland *et al.* [7], but the dominance of *P. flesus* during the early medieval period and the timing of the transition between the species has only now been revealed by applying collagen fingerprinting on these fish remains. This chronological shift between flatfish species is significant for mirroring the gradual transition from freshwater and estuarine exploitation to marine fishing seen more generally during the medieval period. This so-called fish event horizon, is characterized by a relative decrease in freshwater fish exploitation and an increased focus on marine species, such as Gadidae and Clupeidae, probably caused by a multitude of factors such as socio-economic changes, warmer climate and pollution [11]. The results here show that the transition from the more estuarine and riverine living species *P. flesus* to the more marine *P. platessa* during the eleventh century in York coincided with the general intensification of marine fishing in northwest Europe.

The five misidentified samples were thought to be flatfish during the initial selection using osteological methods. These misidentifications show that traditional zooarchaeology can be prone to mistakes even at higher taxonomic levels and that ZooMS is a more reliable and objective method. It also highlights a limitation of this technique however, where at the moment ZooMS is hampered by a lack of good published reference spectra for many fish species and a limited number of species for which peptide biomarkers have been published. By comparing the initial osteological identifications with the results from ZooMS, it seems that traditional morphological methods need to remain at a family level for vertebrae, but selected cranial elements can be (cautiously) identified successfully to species as long as good reference collections are available for consultation. ZooMS can make an important contribution to identify elements for which there are no diagnostic criteria, such as vertebrae (Wouters *et al.* [4]) and fragmented bones, and to clarify cranial elements that are of uncertain species-level attribution.

### 4.3. Other applications

This is only one of a few in-depth studies focusing on a single order of Actinopterygii that have found diagnostic biomarkers for all individual species considered. This shows that ZooMS has much potential in this often overlooked group of animals to identify different taxa. In addition to archaeological applications, these peptide biomarkers provide a cheaper alternative to DNA barcoding approaches used in fisheries management to verify the taxon of fish intended for consumption. Recent studies have indicated that modern day fisheries are still troubled by misidentifications in the food chain of wild-caught fish, including flatfish (e.g. [14–17]). ZooMS could potentially also be applied to answer other ecological questions such as the trophic food webs of flatfish and the ecology of their predators and indeed those of many other species through, for example, gut content analysis (e.g. [63,64]).

## 5. Conclusion

Collagen fingerprinting enables greater depth in the analysis of flatfish remains from European archaeological sites and can improve interpretations of past fisheries, trade and consumption behaviour. Eight collagen peptide markers, described using MALDI-TOF MS and LC-MS/MS, suffice to identify at least 18 different species of flatfish found in European waters. By analysing 202 fish bones from the three archaeological case studies, species previously unreported from the sites became apparent, which showed that there is still an unknown diversity of flatfish in archaeological assemblages. Furthermore, providing a better understanding of species presences through time, major shifts of fisheries can be detected at a detail level that was not possible previously without ZooMS.

ZooMS collagen fingerprinting continues to be of crucial importance to fully understand fish assemblages, and the increasing number of markers available for species identification, will contribute to a more detailed understanding of historical fisheries.

## Data Availability

All necessary data is included in the main body of the paper, through tables and figures. Additional data can be found in the supplementary files. Datafiles of the MALDI-TOF MS spectra, LC-MS/MS raw and mgf files, and MZID files of the Mascot query against the collagen database of the reference samples and the MALDI-TOF MS spectra of the archaeological samples were deposited on Dryad and can be accessed by following this link: https://doi.org/10.5061/dryad.5qfttdz7f [65]. The data are provided in electronic supplementary material [66].
